# A Potential Role of RUNX2- RUNT Domain in Modulating the Expression of Genes Involved in Bone Metastases: An In Vitro Study with Melanoma Cells

**DOI:** 10.3390/cells9030751

**Published:** 2020-03-19

**Authors:** Michela Deiana, Luca Dalle Carbonare, Michela Serena, Samuele Cheri, Simona Mutascio, Alberto Gandini, Giulio Innamorati, Pamela Lorenzi, Michela Cumerlato, Jessica Bertacco, Franco Antoniazzi, Maria Grazia Romanelli, Monica Mottes, Donato Zipeto, Maria Teresa Valenti

**Affiliations:** 1 Department of Medicine, University of Verona, University of Verona, 37100 Verona, Italy; michela.deiana@univr.it (M.D.); samuele.cheri@univr.it (S.C.); mariateresa.valenti@univr.it (M.T.V.); 2 Department of Neurosciences, Biomedicine and Movement Sciences, University of Verona, 37100 Verona, Italy; michela.serena@univr.it (M.S.); simona.mutascio@univr.it (S.M.); pamela.lorenzi@univr.it (P.L.); michela.cumerlato@studenti.univr.it (M.C.); jessica.bertacco@univr.it (J.B.); mariagrazia.romanelli@univr.it (M.G.R.); monica.mottes@univr.it (M.M.); donato.zipeto@univr.it (D.Z.); 3 Department of Surgery, Dentistry, Pediatrics and Gynecology, University of Verona, 37100 Verona, Italy; alberto.gandini@univr.ir (A.G.); giulio.innamorati@univr.it (G.I.); franco.antoniazzi@univr.it (F.A.)

**Keywords:** RUNX2, RUNT domain, PTHrP, bone, metastasis

## Abstract

Ectopic expression of RUNX2 has been reported in several tumors. In melanoma cells, the RUNT domain of RUNX2 increases cell proliferation and migration. Due to the strong link between RUNX2 and skeletal development, we hypothesized that the RUNT domain may be involved in the modulation of mechanisms associated with melanoma bone metastasis. Therefore, we evaluated the expression of metastatic targets in wild type (WT) and RUNT KO melanoma cells by array and real-time PCR analyses. Western blot, ELISA, immunofluorescence, migration and invasion ability assays were also performed. Our findings showed that the expression levels of bone sialoprotein (BSP) and osteopontin (SPP1) genes, which are involved in malignancy-induced hypercalcemia, were reduced in RUNT KO cells. In addition, released PTHrP levels were lower in RUNT KO cells than in WT cells. The RUNT domain also contributes to increased osteotropism and bone invasion in melanoma cells. Importantly, we found that the ERK/p-ERK and AKT/p-AKT pathways are involved in RUNT-promoted bone metastases. On the basis of our findings, we concluded that the RUNX2 RUNT domain is involved in the mechanisms promoting bone metastasis of melanoma cells via complex interactions between multiple players involved in bone remodeling.

## 1. Introduction

Skeletal metastases occur when cancer cells from a primary tumor invade the bone. Generally, bone metastases are associated with breast, prostate and lung cancers [1]. Bone metastases were also found in patients affected by malignant melanoma (MM) [2]. Bone invasion by cancer cells disrupts the balance between osteoblasts and osteoclasts. Therefore, osteoblastic, osteolytic or mixed-bone metastases may result, depending on the phenotype of the target cell [3,4]. However, both osteoblasts and osteoclasts are affected by cancer cells in skeletal metastases. Once cancer cells invade the bone, patient survival chances decrease. In addition, as pathological fractures, pain, hypercalcemia and bone marrow aplasia occur in patients with skeletal metastases, quality of life worsens considerably. Therefore, a multidisciplinary approach aiming to prevent skeletal metastases and identify more effective treatments is necessary [5]. In addition, new studies should be performed to deeply understand which molecular pathways are involved in the interaction between cancer cells and the bone microenvironment. This aim is particularly relevant since the molecular mechanisms involved in the metastatic progression of melanoma are complex. In, M.M.; mutations in transcription regulators, such as BRAF, MITF, KIT, NRAS, PTEN and P53, as well as in TERT, occur frequently [6]. Several studies demonstrated the involvement of RUNX2, the master transcription factor of osteogenic differentiation, in the development of melanoma [7,8]. In fact, besides inducing osteogenic differentiation through mesenchymal stem cell commitment to pre-osteoblasts, RUNX2 is involved in many cellular transformation pathways, such as apoptosis, epithelial–mesenchymal transition (EMT), angiogenesis and metastatic processes [7]. RUNX2 overexpression has been reported in breast cancer, pancreatic cancer, prostate cancer, lung cancer, ovarian epithelial cancer and melanoma. In previous studies, we identified RUNX2 as a stemness marker for cancer [9,10] and observed higher levels of RUNX2 expression in thyroid cancer patients with bone metastases [11]. RUNX2 appears to be involved in the osteolytic process [7,12]. Importantly, the bone sialoprotein (IBSP) and osteopontin (SPP1) coding genes, which are regulated by RUNX2, play important roles in bone metastases derived from osteotropic cancers [13]. In particular, BSP is associated with adhesion, proliferation, invasion, angiogenesis and metastasis [13,14]. Similarly, the *SPP1* (secreted phosphoprotein 1 )gene product, OPN(osteopontin), was observed in bone metastases [15]; it was also reported that reduced expression of SPP1 in melanoma cells is associated with a lower incidence of bone metastases [16]. Importantly, overexpression of parathyroid hormone-related protein (PTHrP) was observed in tumors with metastasized bone tissue [17]. In particular, PTHrP exerts its role in cancer progression and metastases in autocrine (enhancing proliferation, survival and apoptosis resistance), paracrine (inducing RANKL(Receptor Activator of Nuclear Factor Kappa B Ligand) expression in osteoblasts to activate bone resorption) and intracrine (promoting survival, anoikis evasion and cell invasion) manners [17]. PTHrP was demonstrated to be regulated by RUNX2 [18] in head and neck squamous cell carcinoma, and it was also shown that transient exposure to PTHrP increases VEGFR2 expression through pERK stimulation [19]. In addition, RUNX2 promotes esophageal carcinoma by activating the AKT and ERK signaling pathways [20]. Recently, we demonstrated that the RUNT domain, namely the RUNX2 DNA binding domain, is involved in different pathways leading to melanoma transformation [21]. Considering that RUNX2 induces osteogenic genes expression through the RUNT DNA binding domain, we hypothesized that the RUNT domain might also be responsible for the bone tropism of cancer. With this aim, we analyzed the effects of RUNT domain in melanoma cells, focusing on the modulation of metastatic gene expression and the activity of factors that promote osteotropic ability.

## 2. Materials and Methods

### 2.1. Cell Cultures

We used A375 (American Type Culture Collection; ATCC: CTRL-1619TM) and MELHO (DSMZ-Deutsche Sammlung von Mikroorganismen und Zellkulturen) human melanoma cells. The RUNT KO cells were obtained using CRISPR/Cas9 as we previously described [21]. Cell lines were cultured under 5% CO_2_ and in RMPI (1640 (Roswell Park Memorial Institute) growth medium (Sigma-Aldrich, St. Louis, MO, USA) containing 10% fetal bovine serum (FBS) (Sigma-Aldrich), supplemented with antibiotics (1% penicillin/streptomycin) and 1% glutamine. All cell lines were tested negative for mycoplasma using the LookOut Mycoplasma PCR Detection Kit (Sigma-Aldrich).

Once 80% confluence was reached, cells were harvested, washed and counted using a Burker haemocytometer for all experiments.

### 2.2. Construction of RUNX-2 Expression Vector

The RUNX-2 gene was cloned into the pcDNA3 vector as previously described [22,23]. Briefly, the full-length human RUNX-2 open-reading frame (accession number NM_001024630 transcript variant 1) was amplified by polymerase chain reaction (PCR) from the pCMV6 Runx-2 Myc-DDK plasmid (OriGene Technologies, Inc. Rockville, MD, USA#:RC212884,) using the forward primer Runx2F-EcoRV (5′- gcggatatcTTCGCCTCACAAACAACC-3′) and the reverse primer Runx2R-XhoI (5′-ggacctcgagATATGGTCGCCAAACAGAT-3′); underlined nucleotides represent the restriction sites. The amplified fragment was inserted in the pCR^TM^2.1 cloning vector(Invitrogen, Thermo Fischer Scientific, Waltham, MA, USA), then excised by EcoRV/XhoI digestion and finally cloned in pcDNA3-Flag-HA vector (Addgene, Watertown, MA, USA, #10792, Watertown, MA, USA). The cloned fragment was sequenced at the BMR Genomics facility (http://www.bmr-genomics.it). RUNX-2 expression was validated by Western blot.

### 2.3. Exogenous PTHrP Supplementation

The exogenous PTHrp peptide (PeproTech, Rocky Hill, NJ, USA) was added to A375, 3G8, MELHO and 1F5 melanoma cells seeded into 24-well plates at a concentration of 100 µg and incubated for 24 h. Treated cells were then harvested to perform expression analyses.

### 2.4. AKT and ERK Inhibition

A375 and MELHO melanoma cells were plated in 96-well plates at a density of 1000 cells per well and incubated overnight. Cells were then treated with ERK1/2 and AKT inhibitors (SCH772984 and GSK690693, Selleckchem, Houston, TX, USA) for 24 h at a final concentration of 2 µM in RPMI1640 10% FBS. Cultured media were collected to perform ELISA assays, while cells were stored for gene expression analysis.

### 2.5. PCR Array

PCR arrays were performed using a TaqMan™ Human Tumor Metastasis Array (Thermo Fisher Scientific, Waltham, MA USA) according to the manufacturer’s instructions. The amplification reaction and the results analysis were carried out using a QuantStudio™ 3 Real-Time PCR System equipped with QuantStudio^®^ Design and Analysis desktop software (Thermo Fisher Scientific).

### 2.6. Real-Time RT-PCR

Total RNA extraction and RT were performed as previously reported [21]. PCRs were performed in a total volume of 25 µl using 20 ng of cDNA for each sample. Real-time PCR was performed using TaqMan Universal PCR Master Mix (Thermofisher Corporation, Waltham, MA, USA) and TaqMan pre-designed probes for each gene (*VEGFA*, Hs00900055_m1; *VEGFR*, Hs01052961_m1; *CD31*, Hs01065279_m1; *IBSP*, Hs00173720, *OPN*, Hs00167093_m1). Gene expression for *MMP9* (FW AGACCTGGGCAGATTCCAAAC, RV CGGCAAGTCTTCCGAGTAGT, Sigma-Aldrich) was tested using the Power SYBR^®^ Green PCR Master Mix (Thermo Fisher Scientific). Gene expression was normalized to the housekeeping β2-microglobulin (*β2M*, Hs99999901_s1) gene, and the relative fold expression differences were calculated. TaqMan SDS analysis software was used to analyze the Ct values. Three independent experiments with three replicates for each sample were performed.

### 2.7. Western Blot Analysis

RIPA buffer was used for protein extraction (Thermo Fisher Scientific) and protein concentrations were determined by BCA assay (Thermo Fisher Scientific). Protein samples were separated by sodium dodecyl sulfate-polyacrylamide gel electrophoresis (SDS-PAGE) using mini-PROTEAN^®^ TGXTM precast gradient 4–20% gels (BioRad, Hercules, CA, USA) and transferred onto polyvinylidene difluoride (PVDF) membranes (Thermo Fisher Scientific). PVDF membranes were then probed with the primary and secondary antibodies reported in Table 1.

Signals were detected using a chemiluminescence reagent (ECL, Millipore, Burlington, MA, USA), and images were acquired using an LAS4000 Digital Image Scanning System (GE Healthcare, Little Chalfont, UK). Densitometric analyses were performed using the ImageJ software, and the relative protein band intensity was normalized to β-actin and expressed as the optical density (OD) ratio. The data were obtained from three independent experiments.

### 2.8. Immunofluorescence

Cells were fixed and processed according to the manufacturer’s protocols. BSP primary antibodies (Abcam, Cambridge, UK) were diluted (as reported in the datasheet) in Antibody Dilution Buffer and incubated overnight at 4 °C. The slides were then incubated with the Alexa Fluor^®^ 488 anti-rabbit (Cat. #A-11034) secondary antibody, and nuclear staining was performed by using ProLong™ Gold Antifade Mountant with DAPI (Thermo Fisher Scientific). Images were captured using a Leica DM2500 microscope (Leica Microsystem, Wetzlar, Germany). In particular, four different fields were measured for each sample in three independent experiments, and each field contained approximately 80–100 total cells.

### 2.9. Migration to Bone Ability

To assess bone tropism, we first compared cells’ ability to migrate in the presence or absence of a bone fragment. Therefore, cells were seeded on a 6-well plate at a density of 500,000 per well. After adhesion, half of each well was scratched using a cell scraper, and the relative migration distance (RMD) was calculated in the absence or presence of a bovine bone slice (Nøddevænget 3, DK-7300 Jelling, Denmark) placed at the same distance in all samples. Cultures were carried out for 2 days using DMEM (Dulbecco’s Modified Eagle Medium) supplemented with 10% FBS and Glutamax (all from Thermo Fisher Scientific) at 37 °C and 5% CO. The migration ability assay was conducted with an EVOS™ FL Auto Imaging System (Thermo Fisher Scientific) under time-lapse protocol for 48 h. Distances between the cell front and the bone slice or the signed blank space for every well were measured at the beginning and at the end of each experiment. Relative migration distances (RMDs) were calculated using the following formula: RMD = (t0–t1)/t0, where t0 is the distance between the cell front and the bone slice at time zero of the assay and t1 is the same distance at the end, as previously reported [21]. The RMD of WT cells was normalized to the RMD of RUNX2 KO cells to evaluate the role of RUNX2 in migration in the presence or absence of bone fragments. In addition, we performed experiments with the transwell system to further analyze migration and evaluate invasion ability, as previously described [24]. For both migration and invasion assays, 1 × 10^4^ cells were seeded onto the upper chamber of transwell plates of 8 µm diameter (Corning Incorporated, NY) in the presence of RPMI supplemented with 1% FBS for 24 h (migration assay) or 48 h (invasion assay). The invasion assays were performed by coating the upper chamber with Matrigel. The lower chamber was filled with medium with or without bone fragments (Noddevaenget 3, DK-7300 Jelling, Denmark). After 48 h, cells adherent to the upper surface of the membrane were removed. Thereafter, cells in the membrane underside were fixed with 4% of paraformaldeide and stained with DAPI(4′,6-diamidino-2-phenylindole). Cells were then visualized under a Leica DM 2500 (Leica Microsystem, Wetzlar, Germany) to take pictures and to evaluate the number of adherent cells. Cells were counted in ten random fields at 40X magnification.

### 2.10. ELISA

For PTHrP protein detection, we performed an ELISA (Fine Biotech Co Ltd., Wuhan, China). WT and RUNT KO cell lines were plated onto 96-well plates at a density of 10,000 cells/well. After 3 days of culture, the medium was collected and centrifuged at 1000 g for 20 min at 4 °C. Standards were prepared following the manufacturer’s instructions. Samples and standards were plated into the ELISA microplate, and the assay was conducted according to the manufacturer’s instructions.

### 2.11. Bioinformatics Analysis

RUNX2, PTHrP, AKT and ERK proteins were submitted to the STRING portal (https://string-db.org) for independent inspection related to their predicted connections.

### 2.12. Statistical Analysis

Student’s paired t-test was used to compare the variation of variables between two groups. Differences were considered statistically significant at *p* < 0.05. Experiments were performed at least three times. Statistical analyses were performed using SPSS (Statistical Package for Social Science) for Windows, version 16.0 (SPSS Inc., Chicago, IL, USA).

## 3. Results

### 3.1. RUNX2 RUNT Domain Empowers RUNX2 Metastatic Ability in Melanoma Cells

**Figure 1:**
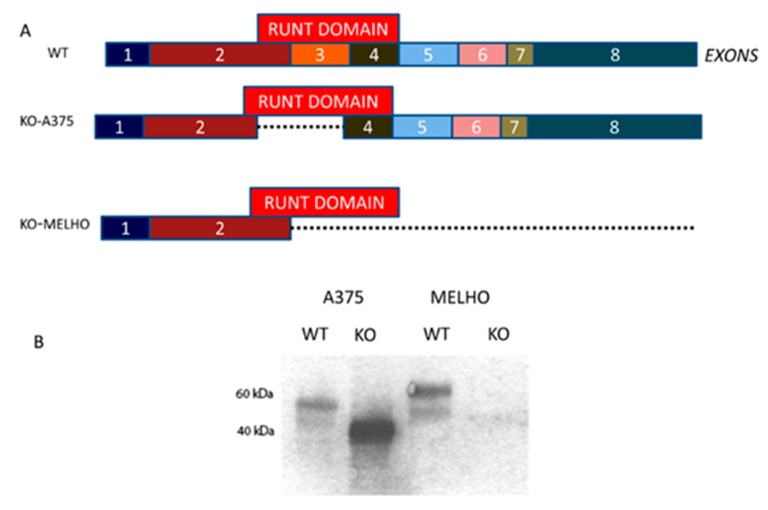
(**A**) Location of the RUNT coding domain within RUNX2 cDNA as reported in RefSeq NP 00101019801.3 (**B**) Western blot showing RUNX2 in A375, KO-A375, MELHO and KO-MELHO melanoma cells.

Figure 1A shows a schematic representation of the RUNT domain coding region within the RUNX2 cDNA. By using the CRISPR/Cas9 technology, we partially deleted the RUNT domain or knocked out the whole RUNX2 gene containing the RUNT domain in A375 and MELHO melanoma cells (Figure 1A). Therefore, we obtained a lower and null RUNX2 proteins in KO-A375 and KO-MELHO, respectively (Figure 1B).

**Figure 2:**
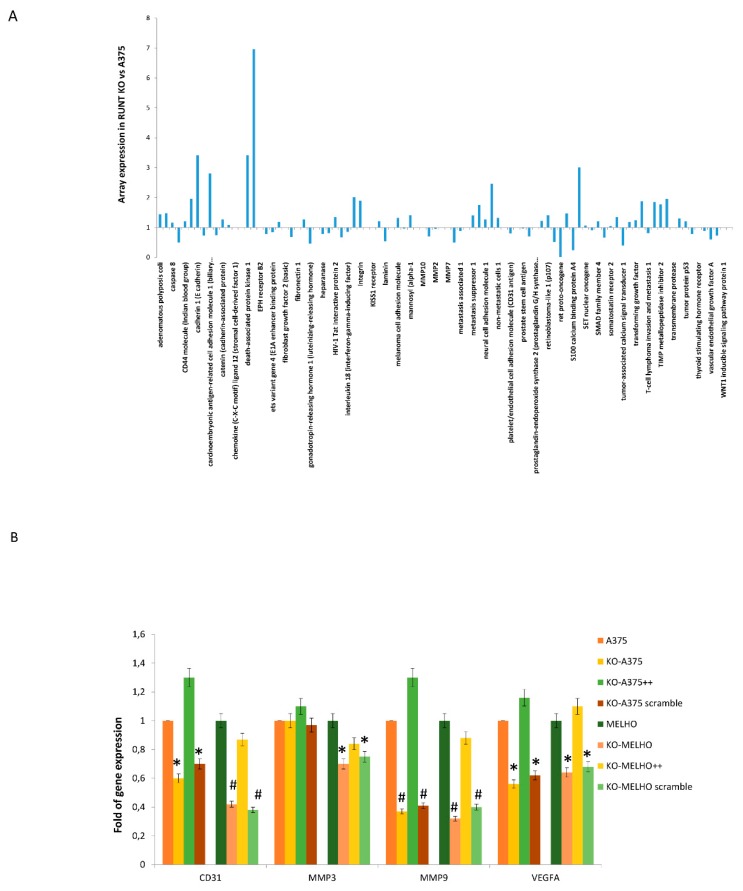
(**A**) Array PCR: Expression levels of metastatic genes evaluated by TaqMan™ Human Tumor Metastasis Array. (**B**) The downregulation of gene expression in RUNT-KO cells compared to wild type (WT) melanoma cells confirmed the array results. (* *p* < 0.05; # *p* < 0.01).

Then, we evaluated the metastatic gene expression profile in A375 and RUNX2 RUNT KO (RUNT KO) melanoma cells by using a Human Tumor Metastasis Array. The data showed lower expression of several genes involved in the metastatic process in RUNT KO cells compared to A375 cells (Figure 2A and Table S1). To validate these findings, we performed real-time PCR assays for four selected lower expressed genes, namely, platelet and endothelial cell adhesion molecule 1 (CD31), matrix metallopeptidase 3 (MMP3), matrix metallopeptidase 9 (MMP9) and vascular endothelial growth factor A (VEGFA) in WT (A375 and MELHO) and RUNT-KO (KO-A375 and KO-MELHO) melanoma cells. The investigated genes were downregulated in both RUNT-KO cell lines, with the exception of MMP3 expression, which was unchanged in KO-A375 (Figure 2B). Gene expression was restored in all KO cells upon resetting the RUNT domain (Figure 2B). Similarly, expression levels of CD31 and VEGFA, which are genes involved in metastatic processes, were reduced in RUNT KO cells (Figure 2B), thereby confirming the array data.

**Figure 3:**
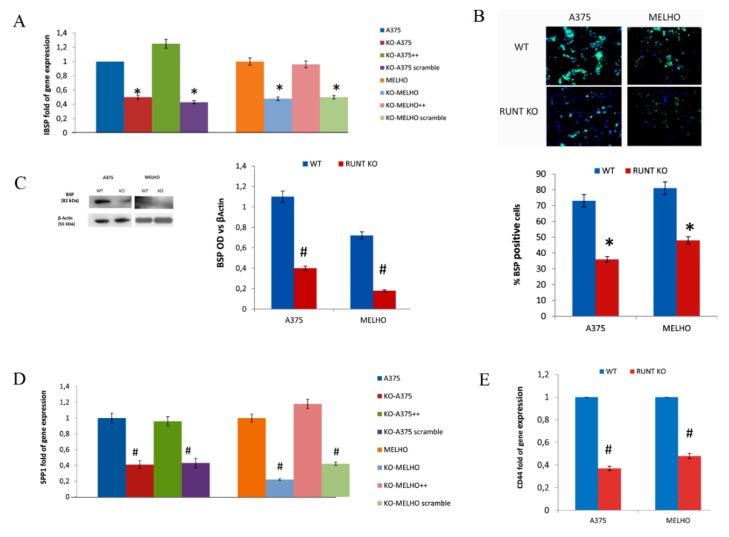
(**A**) Gene expression of the bone metastatic gene *IBSP* is lower in RUNT KO cells than in WT melanoma cells. Accordingly, there is a lower percentage of BSP-positive cells (**B**) and lower levels of the BSP protein (**C**) in RUNT KO cells compared to WT melanoma cells. Gene expression levels of the bone metastatic genes *SPP1* (**D**) and *CD44* (**E**) are lower in RUNT KO cells than in WT melanoma cells. (* *p* < 0.05; # *p* < 0.01); magnification 40X.

We then tested RUNT domain influence in driving melanoma cell migration to the bone by analyzing the expression of IBSP and SPP1 genes. As shown in Figure 3A, the expression levels of IBSP decreased in RUNT-KO cells. We also observed a reduced number of BSP-positive cells (Figure 3B) as well as reduced BSP protein expression in RUNT-KO cells (Figure 3C) compared to WT cells. SPP1 gene expression levels were also reduced in RUNT-KO cells (Figure 3D), which was rectified by RUNT re-expression. Interestingly, the expression of CD44, an osteopontin receptor which is considered a stemness marker in several kinds of cancers, was also reduced in RUNT-KO cells (Figure 3E).

### 3.2. The RUNT Domain Increases PTHrP Levels in Melanoma and Activates AKT and ERK Pathways

**Figure 4:**
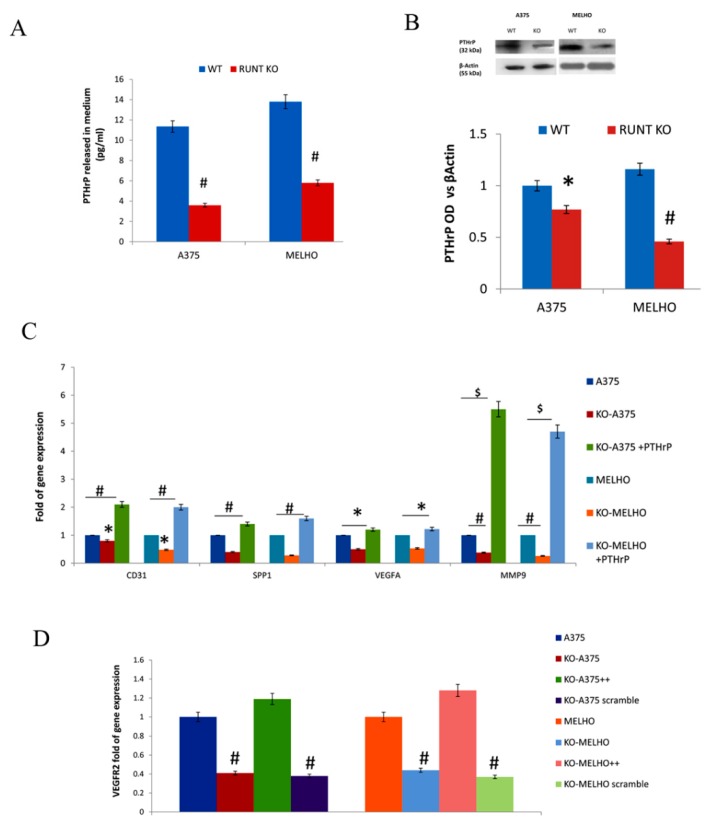
Both released (**A**) and intracellular (**B**) levels of parathyroid hormone-related peptide (PThrP) are lower in RUNT KO cells than in WT cells. The addition of exogenous PTHrP restores gene expression levels in both KO-cell lines (**C**). (**D**) VEGFR2 gene fold expression is lower in RUNT-KO cells than in WT cells. Re-expression of the RUNT domain restores VEGFR2 gene expression in both cell lines. (* *p* < 0.05; # *P* < 0.01; $ *p* < 0.001).

As PTHrP expression is regulated by RUNX2, we measured PTHrP levels in WT and RUNT-KO melanoma cell culture media. Interestingly, we observed a significant reduction in PTHrP concentration in RUNT KO cells media compared to the WT cell media (Figure 4A), as well as a reduction in intracellular PTHrP levels (Figure 4B) in both cell lines.

In order to confirm the RUNT domain role in inducing PTHrP expression, we cultured KO cells in the presence of exogenous PTHrP (+exPTHrP). As shown in Figure 4C, all investigated genes related to metastatic ability were upregulated in KO cells treated with exogenous PTHrP. In addition, we analyzed the expression of VEGFR2, which is associated with increased expression of PTHrP in other cancers. As shown in Figure 4D, downregulation of VEGFR2 expression was observed in RUNT KO cells. Restoration of the RUNT domain re-established VEGFR2 gene expression.

**Figure 5:**
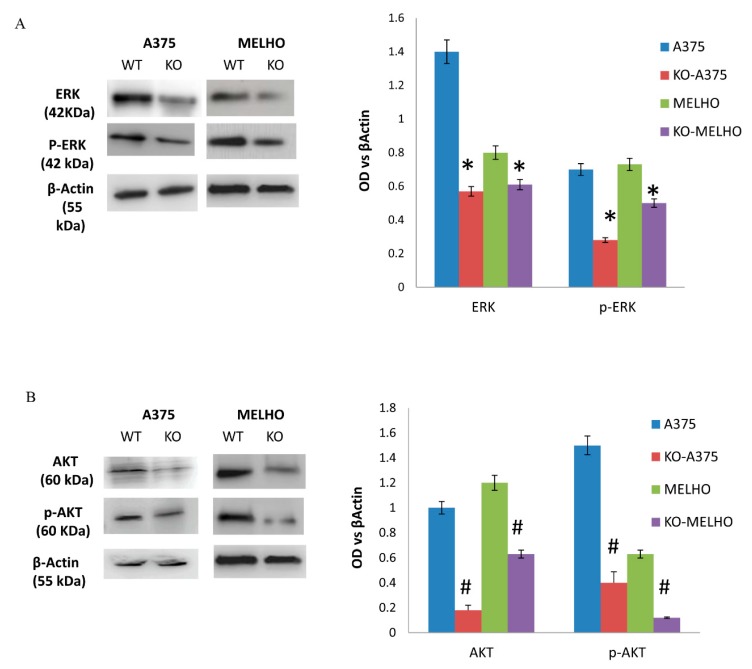
The expression of both ERK (**A**) and AKT (**B**) as well as the expression of the related phosphorylated proteins is lower in RUNT KO cells than in WT cells (* *p* > 0.05; # *p* > 0.01).

Considering the modulatory role of VEGFR2 in the ERK pathway, we then looked for ERK pathway modifications in KO cells. The observed reduced levels of ERK and pERK proteins expression in RUNT KO cells compared to WT cells suggested an activating role of the RUNT domain (Figure 5A). ERK and AKT pathways are strongly associated with oncogenic transformation, therefore we investigated the role of the RUNT domain in AKT pathway modulation. As shown in Figure 5B, protein expression levels of both AKT and pAKT were lower in RUNT KO cells than in WT cells in both cell lines.

### 3.3. RUNX2 Regulates AKT and ERK in a Reciprocal Way

**Figure 6:**
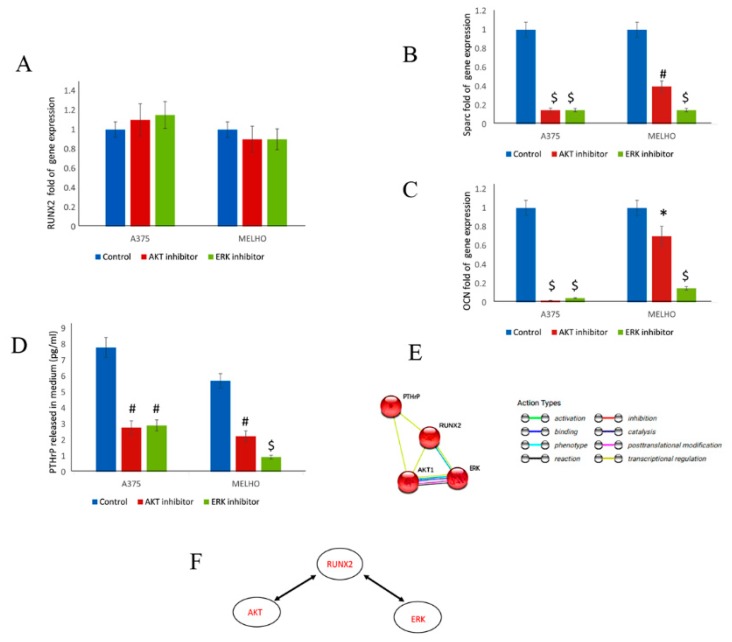
RUNX2 gene expression is not affected by AKT or ERK inhibitors (**A**) in WT melanoma cells. However, downstream target genes of RUNX2, namely Osteonectin (SPARC) (**B**) and Osteocalcin (OCN) (**C**) are downregulated by AKT or ERK inhibitor treatment. Both AKT and ERK inhibitors reduce the amount of PTHrP released by WT melanoma cells (**D**). Bioinformatics analyses show the interactions occurring (**E**). The reciprocal interaction and activation of RUNT/RUNX2 with AKT and ERK signaling (**F**). (* *p* < 0.05; # *P* < 0.01; $ *p* < 0.001).

To evaluate the interaction between RUNT and AKT/ERK signaling pathways, we treated WT cells with either AKT or ERK inhibitors. Our data showed that the inhibition of AKT and ERK pathways did not affect RUNX2 gene expression (Figure 6A). However, the inhibition of the AKT and ERK pathways heavily reduced the expression of RUNX2-downstream genes, namely SPARC (Osteonectin), and OCN (Osteocalcin), thus demonstrating the effects of their inhibition on RUNX2 transcriptional activity (Figure 6B,C).

Importantly, inhibition of both the AKT and ERK pathways reduced the amount of PTHrP released in the WT melanoma cell medium (Figure 6D). Accordingly, bioinformatic analyses showed a fine interaction between these molecular factors (Figure 6E). For the first time in melanoma, we also observed interaction and reciprocal activation between RUNT/RUNX2 and AKT/ERK signaling (Figure 6F).

### 3.4. Osteotropism is Reduced in RUNT KO Melanoma Cells

**Figure 7:**
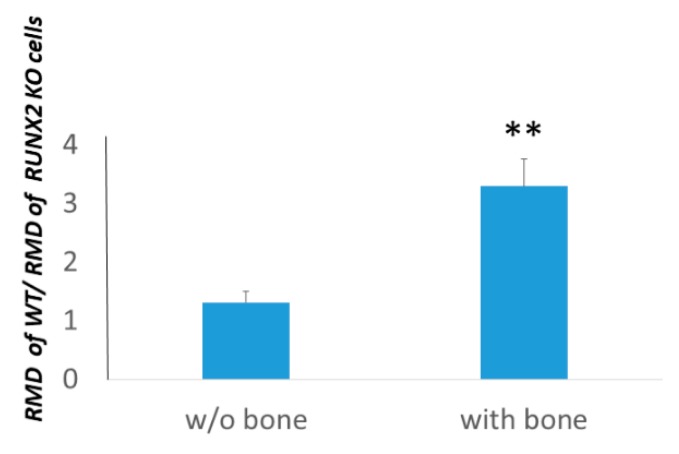
Relative migration distance ratio between WT and related RUNT KO cells. The presence of the RUNT domain in melanoma cells (WT) appears significantly more effective in promoting migration when bone fragments are present. ** *p* < 0.01.

At first, we evaluated the migration ability of cells either in the presence or in absence of bone fragments in vitro. In particular, we calculated the levels of the relative migration distance of WT versus RUNX2 KO cells in the presence or absence of bone fragments. We observed that RMD levels of, W.T.; normalized with the RMD of RUNX2 KO cells, were higher in the presence of bone fragments (Figure 7). Therefore, the expression of the RUNT domain increases more cell migration in the presence of bone.

**Figure 8:**
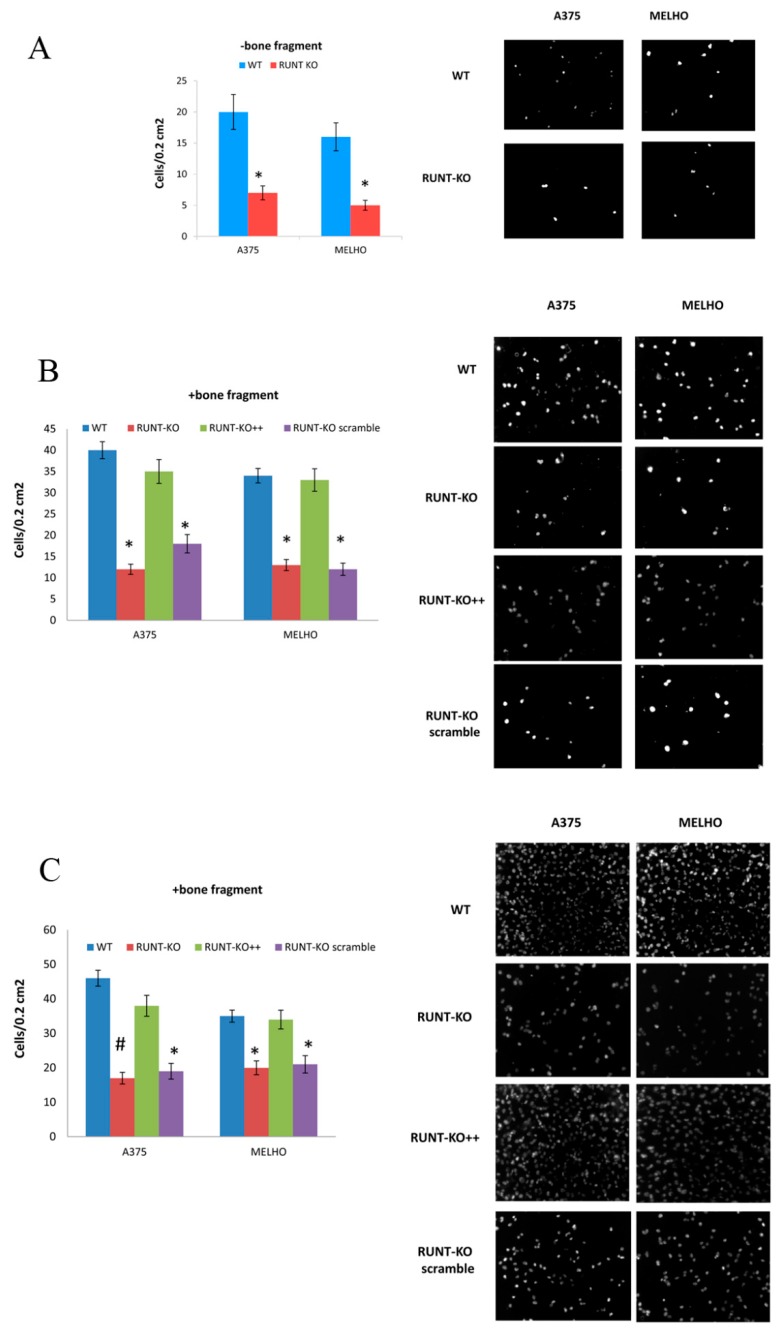
The ability to migrate was higher in WT compared to RUNT-KO cells (**A**). In addition, the presence of bone fragments increased migration (**B**) and invasion (**C**) abilities, which were greater in WT compared to RUNT-KO cells. Restored expression of RUNT domain in RUNT-KO cells re-established these abilities. Magnification 10X (* *p* > 0.05; # *p* > 0.01).

To further analyze the role of the RUNT domain in driving melanoma cell migration to bone fragments, we tested the migration and invasion ability of WT and RUNT-KO melanoma cells, respectively, in a transwell system. The migration ability in the absence of bone fragments was lower for RUNT-KO cells compared to WT (Figure 8A). In the presence of bone fragments, the ability to migrate was strongly increased in WT cells, while a comparatively lower ability to migrate was recorded for RUNT-KO cells (Figure 8B). However, the ability to migrate was restored in RUNX2-transfected RUNT KO cells (RUNT-KO++) (Figure 8B). Similarly, the invasion ability was higher in WT compared to RUNT-KO cells and the re-expression of RUNT restored this ability in both RUNT-KO cell lines (Figure 8C).

## 4. Discussion

The bone microenvironment regulates complex and relevant processes such as hematopoiesis, osteogenesis and osteolysis [1]. Various studies demonstrated that different molecular mechanisms are involved in promoting cancer cells residency in bone through chemotaxis in bone niches [25]. Most bone metastases occur when prostate, breast and lung primary tumors spread to the bone [26,27]. However, other primary tumors can induce bone metastases [27]. A retrospective study evaluating 98 MM patients reported that bone metastases occurred in 17.3% of cases [2,28]. Recently, radiographic imaging revealed the presence of bone metastases in 4.1% of patients at all stages of MM and in 17.2% of MM patients with metastatic disease [29]. However, as isotope bone scans may produce false-negative results [30], the actual frequency of bone metastases in metastatic MM should be considered to be higher. In post-mortem studies, bone metastases were found in 48.6% of patients with metastatic MM [31].

Transcription factor RUNX2 is the master gene of osteogenic differentiation. Its expression is high in pre-osteoblasts and in early osteoblasts, but decreases in mature osteoblasts [32]. However, RUNX2 is involved also in cellular transformation and appears upregulated in different solid tumors [9]. RUNX2 is ectopically expressed in melanoma and plays an important role in progression [7,8,33,34]. It belongs to the RUNX family, which includes also RUNX1 and RUNX3. These are RUNT-related heterodimeric transcription factors consisting of a DNA-binding “A” subunit and a non-DNA-binding “B” subunit, which enhances its affinity with DNA [35]. Among other domains, RUNX2 protein has a conserved 128-amino acid RUNT domain encoded by exons 2 to 4, which is necessary for DNA binding and heterodimerization with the non-DNA binding “B” subunit [32].

RUNX2 involvement in regulating the epithelial–mesenchymal transition (EMT) process has been demonstrated in melanoma [14]. Recently, we demonstrated that the RUNX2 RUNT domain affects EMT by increasing the expression of N-cadherin and reducing the expression of E-cadherin [21]. In addition, we found that the RUNT domain also promotes EMT by upregulating the expression of vimentin [21]. This is a noteworthy finding, considering vimentin correlation with high tumor growth rate, invasiveness and poor prognosis [35].

In lung cancers, RUNX2 overexpression was shown to promote EMT via direct regulation of vimentin along with other proteins [36,37]. Recently, it was demonstrated that RUNX2 participates in the transcriptional regulation of vimentin [38]. Yet, specific mechanisms related to RUNX2-dependent transcriptional regulation of vimentin need to be further explored in detail.

We demonstrated that the RUNT domain promotes melanoma cell proliferation and migration [21]. Accordingly, being aware of the strong association between RUNX2 and bone remodeling agents, we hypothesized that the RUNX2 RUNT domain is involved in mechanisms that promote bone metastasis in melanoma cells. With the aim to understand the molecular mechanisms modulated by RUNX2 RUNT domain in promoting bone metastasis, we performed gene expression analyses and cell signaling investigations in vitro. We obtained RUNT KO melanoma cell lines using CRISPR/Cas9-mediated gene editing. In RUNT KO melanoma cells, reduced expression of genes involved in metastatic processes (e.g., CD31, MMP9 and VEGFA) was observed, and genes involved in promoting bone metastasis, such as IBSP (coding for bone sialoprotein) and SPP1 (coding for osteopontin) [15], were downregulated. Notably, in RUNT KO melanoma cells, the expression of CD44 was also reduced. CD44 was identified as a receptor for hyaluronic acid, as well as for osteopontin, collagens and matrix metalloproteinases [39]. CD44 is a stemness marker associated with cancer metastatic progression [39]. The intracellular domain of CD44 can act as a co-transcription factor for RUNX2, inducing MMP-9 expression in breast carcinoma cells [40]. Recently, Senbanjo et al. demonstrated that RUNX2 complexes with the CD44 intracellular domain, thus inducing the expression of metastasis-related genes and increasing migration ability, as well as the formation of tumorspheres in prostatic cancer cells [41]. Notably, targeting of osteopontin or its receptors (e.g., CD44 and Integrin αvβ3) was suggested as a strategy to reduce carcinogenesis [42]. Gupta and coworkers demonstrated that Integrin αvβ3 and CD44 are involved in prostate cancer patients bone loss by promoting osteoclastogenesis through the RUNX2/Smad 5/receptor activator of NF-κB ligand pathways [43].

We also observed a reduced expression of PTHrP, an autocrine/paracrine ligand involved in malignancy-induced hypercalcaemia and skeletal metastatic lesions, in RUNT KO cells [17]. Interestingly, the addition of exogenous PTHrP in KO-cells restored the expression of metastatic genes. It was demonstrated that ectopic expression of RUNX2 increases PTHrP expression in neck and lung cancers [18]. In addition, since the ERK/pERK and AKT/pAKT pathways are associated with both RUNX2 and PTHrP expression, we investigated the modulation of these pathways in WT and RUNT KO melanoma cells. Interestingly, we found that both ERK/pERK and AKT/pAKT signaling processes were affected, entailing lower expression in RUNT KO melanoma cells. These findings suggested that the RUNT domain is involved in PTHrP expression through the regulation of these pathways, thus promoting bone metastasis. Bioinformatic analyses confirmed the RUNX2 interaction with AKT and ERK signalling. However, for the first time, we demonstrated that a reciprocal activation between the RUNX2 and AKT/ERK pathways occurs in melanoma. In fact, by treating WT melanoma cells with AKT or ERK inhibitors, we observed reduced activity of RUNX2, although RUNX2 gene expression was not affected. RUNX2 activity reduction due to inhibition of AKT or ERK signaling decreased PTHrP production in melanoma cells in turn. Therefore, we concluded that RUNX2 is involved in mechanisms that promote bone metastasis.

To evaluate the involvement of RUNX2 in osteotropic mechanisms, we performed in vitro experiments by using bone fragments as previously described by Mannavola and coworkers [24]. Employment of bone fragments was also reported by Templeton and coworkers to evaluate bone colonization of breast cancer cells [44]. Our data showed that RUNX2 KO cells exhibited statistically significant reductions in migration and invasion to bone fragments compared to WT cells. The re-expression of RUNT by transfecting KO cells with RUNX2 expression vectors restored cells’ ability to migrate and invade bone fragments. This finding supports the transcriptional role of the RUNT domain in binding its downstream target genes, such as IBSP and SPP1, which are involved in bone metastasis promotion [45,46]. As discussed above, we observed reduced expression of SPP1 in RUNX2 KO cells. Osteopontin, an SPP1 gene product, was demonstrated to be involved in different steps of carcinogenesis, such as cell mobility, neoangiogenesis, invasion, intravasation and extravasation, as well as bone metastasization [45,47]. In summary, our findings, including reduced osteopontin, bone sialoprotein and CD44, supported RUNX2 involvement in promoting melanoma cell osteotropism. One limitation of our study may be the lack of an in vivo model. However, the purpose of this study was to deepen our understanding of the role of RUNX2 in modulating the molecular mechanisms that promote bone metastasis. Our results showed that RUNX2 is involved in the expression modulation of key genes. SPP1, bone sialoprotein and molecules that enhance bone metastasis, such as PTHrP, are significant examples which could stimulate further studies. In particular, the employment of animal models could be helpful to evaluate the role of the immune system or the angiogenesis process in the metastatic niche.

Finally, since our results showed that the RUNT domain affects the expression and the activity of various molecules involved in bone metastasis, we conclude that RUNX2, via the RUNT domain, may promote bone metastasis of melanoma through a complex scenario affecting different and strongly associated pathways.

## Figures and Tables

**Table 1:** Antibodies used in this study.

Antibody	Ab Dilution	Origin	Secondary Antibody
BSP (Bone Sialoprotein) II	1:1000	(Cell Signaling, 5486)	Anti-rabbit (Cell Signaling, 7074)
AKT (C67E7)	1:1000	(Cell Signaling, 4691)	Anti-rabbit (Cell Signaling, 7074)
p_AKT (193H12)	1:1000	(Cell Signaling, 4058)	Anti-rabbit (Cell Signaling, 7074)
ERK (13F5)	1:1000	(Cell Signaling, 4695)	Anti-rabbit (Cell Signaling, 7074)
p_ERK (D13.14.4E)	1:2000	(Cell Signaling, 4370)	Anti-rabbit (Cell Signaling, 7074)
PTHrP (1D1)	1:1000	(SantaCruz Biotech., Dallas, TX, USA)	Anti-mouse (Cell Signaling, 7076)
β ACTIN (BA3R)	1:5000	(Thermo Scientific)	Anti-mouse (Cell Signaling, 7076)

## Supplementary Materials

The following are available online at https://www.mdpi.com/2073-4409/9/3/751/s1, Table S1: List of modulated genes in RUNT-KO.
