# Supplementary material for: A Potential Role of RUNX2- RUNT Domain in Modulating the Expression of Genes Involved in Bone Metastases: An In Vitro Study with Melanoma Cells
Source: Cells. 2020 Mar 19;9(3):751. doi: 10.3390/cells9030751 (PMC7140624; doi:10.3390/cells9030751)
Supplement: Supplementary file 1 [file cells-09-00751-s001.pdf]

Table S1. List of modulated genes in RUNT-KO

Gene name	Fold of expression in RUNT-KO WT
adenomatous polyposis coli	1.44
breast cancer metastasis suppressor 1	1.48
caspase 8	1.16
cadherin 1 (E cadherin)	3.41
cyclin-dependent kinase inhibitor 2A (melanoma	0.73
carcinoembryonic antigen-related cell adhesion molecule 1 (biliary glycoprotein)	2.81
C-terminal binding protein 1	0.74
catenin (cadherin-associated protein)	1.27
cathepsin K	1.08
chemokine (C-X-C motif) ligand 12 (stromal cell-derived factor 1)	1
chemokine (C-X-C motif) receptor 4	1
death-associated protein kinase 1	3.41
deleted in colorectal carcinoma	6.96
EPH receptor B2	1
v-erb-b2 erythroblastic leukemia viral oncogene homolog 2	0.78
ets variant gene 4 (E1A enhancer binding protein	0.84
FAT tumor suppressor homolog 1 (Drosophila)	1.19
fibroblast growth factor 2 (basic)	1
fibroblast growth factor receptor 4	0.68
fibronectin 1	1
FXYD domain containing ion transport regulator 5	1.27
gonadotropin-releasing hormone 1 (luteinizing-releasing hormone)	0.46
hepatocyte growth factor (hepapoietin A; scatter factor)	1
heparanase	0.78
v-Ha-ras Harvey rat sarcoma viral oncogene homolog	0.81
HIV-1 Tat interactive protein 2	1.35
insulin-like growth factor 1 (somatomedin C)	0.67
interleukin 18 (interferon-gamma-inducing factor)	0.85
interleukin 1	2.01
integrin	1.89
KiSS-1 metastasis-suppressor	1
KISS1 receptor	1
v-Ki-ras2 Kirsten rat sarcoma viral oncogene homolog	1.21
laminin	0.54
LY6/PLAUR domain containing 3	1
melanoma cell adhesion molecule	1.32
met proto-oncogene (hepatocyte growth factor receptor)	0.96
mannosyl (alpha-1)	1.41
MMP1	1
MMP10	1
MMP14	0.7
MMP2	0.95
MMP3	1
MMP7	1
MMP10	0.5
metastasis associated 1	0.88
metastasis associated 1 family	1
metastasis suppressor 1	1.4
v-myc myelocytomatosis viral oncogene homolog (avian)	1.75
neural cell adhesion molecule 1	1.27

neurofibromin 2 (merlin)	2.46
non-metastatic cells 1	1.32
nuclear receptor subfamily 4	1
platelet/endothelial cell adhesion molecule (CD31 antigen)	0.8
pinin	1
prostate stem cell antigen	0.97
phosphatase and tensin homolog (mutated in multiple advanced cancers 1)	0.7
prostaglandin-endoperoxide synthase 2 (prostaglandin G/H synthase and cyclooxygenase)	1
retinoblastoma 1 (including osteosarcoma)	1.22
retinoblastoma-like 1 (p107)	1.41
retinoblastoma-like 2 (p130)	0.51
ret proto-oncogene	0.01
ras homolog gene family	1.47
S100 calcium binding protein A4	0.24
serpin peptidase inhibitor	3.01
SET nuclear oncogene	1.06
SMAD family member 2	0.91
SMAD family member 4	1.2
synuclein	0.66
somatostatin receptor 2	1.05
spleen tyrosine kinase	1.34
tumor-associated calcium signal transducer 1	0.39
transcription factor 20 (AR1)	1.18
transforming growth factor	1.24
T-cell lymphoma invasion and metastasis 1	0.81
TIMP metallopeptidase inhibitor 1	1.85
TIMP metallopeptidase inhibitor 2	1.77
TIMP metallopeptidase inhibitor 4	1.95
transmembrane protease	1
tumor necrosis factor (ligand) superfamily	1.3
tumor protein p53	1.2
trophoblast glycoprotein	0.78
thyroid stimulating hormone receptor	1
twist homolog 1 (acrocephalosyndactyly 3; Saethre-Chotzen syndrome) (Drosophila)	0.89
vascular endothelial growth factor A	0.6
vascular endothelial growth factor C	0.73
WNT1 inducible signaling pathway protein 1	1
